# Hepatitis B Virus Genotype B and High Expression of Interferon Alpha Receptor β Subunit are Associated With Better Response to Pegylated Interferon Alpha 2a in Chinese Patients With Chronic Hepatitis B Infection

**DOI:** 10.5812/hepatmon.6173

**Published:** 2012-05-30

**Authors:** He Bin Fan, Ya Bin Guo, You Fu Zhu, An Shen Chen, Mu Xiu Zhou, Zhi Li, Li Tong Xu, Xiao Ju Ma, Fu Ming Yan

**Affiliations:** 1 Department of Infectious Disease, The People’s Liberation Army 161 Hospital, Wuhan, China; 2 Department of Infectious Disease, Nanfang Hospital, Southern Medical University, Guangzhou, China; 3 Department of Pathology, The People’s Liberation Army 161 Hospital, Wuhan, China

**Keywords:** Hepatitis B, Chronic, IFNAR2 Protein, Human, Peginterferon alfa-2a

## Abstract

**Background:**

Hepatitis B virus (HBV) is one of leading causes of various hepatic diseases including acute and chronic hepatitis, cirrhosis, and hepatocellular carcinoma. Hundreds of million people worldwide are infected by HBV, chronically.

**Objectives:**

This study in conducted to investigate the inﬂuence of Hepatitis B virus (HBV) genotypes and type I IFN-αreceptor β subunit (IFNAR2) expression in liver on response to treatment with pegylated IFN-α-2a (Peg-IFN-α-2a) for chronic hepatitis B infection.

**Patients and Methods:**

In this study, 65 eligible patients with chronic hepatitis B disease were enrolled. HBV genotypes of these patients were analyzed by using PCR-RFLP of the surface gene of HBV. The expression of IFNAR2 in the liver was immune histochemically investigated using anti-IFNAR2 antibody. All immune histochemical slides were read semi-quantitatively by image analysis. Chronic hepatitis B patients were treated with Peg-IFN-α2a therapy for a 48-week period and followed up for 24 weeks. Baseline characteristics and sustained viral response (SVR) to Peg-IFN-α-2a therapy were evaluated.

**Results:**

55 % of patients exhibited HBV genotype B and 31.7 % patients exhibited HBV genotypes C infections. After treatment with Peg-IFN-α-2a, SVR was achieved in 66.7 % of patients with HBV genotype B and in 26.3 % of patients with HBV genotype C (P = 0.009). Semiquantitative and the image analysis indicated by gray level values revealed a higher IFNAR2 expression in the group with severe inﬂammation (P < 0.001). Patients’ high IFNAR2 protein expression had a signiﬁcant impact on SVR to Peg-IFN-α-2a therapy (P = 0.028).

**Conclusions:**

HBV genotype B and high expression of IFNAR2 in the liver of chronic hepatitis B patients are closely associated with better response to Peg-IFN-α-2a therapy in chronic hepatitis B disease.

## 1. Background

Hepatitis B virus is one of leading causes of various hepatic diseases including acute and chronic hepatitis, cirrhosis, and hepatocellular carcinoma. Hundreds of million people worldwide are infected by hepatitis B virus (HBV), chronically. HBV infection is one of the most common viral infections in China. The seroepidemiological survey on HBV infection conducted in 2006 showed that hepatitis B surface antigen (HBsAg) carrier rate was 7.18 % in all population with an estimated 93 million HBsAg carriers [1]. Antiviral drugs can be used to inhibit HBV replication and thus reduce the risk of progression to cirrhosis, or liver failure or cancer. Currently, two groups of drugs approved by Federal Drug Administration for treatment of chronic hepatitis B disease consist of the immune modulators such as conventional interferon alpha (IFN-α) and pegylated interferon alpha-2a (Peg-IFN-α-2α), and nucleoside–nucleotide analogs such as lamivudine and adefovir dipivoxil. However, not all patients with chronic hepatitis B infection respond to these treatments. IFN-α elicits antiviral, immune modulatory, anti-proliferative, and gene induction properties through binding to type I interferon receptor (IFNAR) [2]. The effect of treatment of chronic hepatitis B disease with IFN-α is sustained viral suppression with HBeAg seroconversion resulted in improved clinical outcomes. Factors known to inﬂuence the response include serum HBV DNA level, alanine aminotransferase (ALT), age , grade of inﬂammatory activity in liver, and host factorsthat might be genetic (sex, cytokine polymorphism). However, IFN-α treatment has several disadvantages including tediousness and requirement of daily injections with high occurrence of side-effects. In order to reduce side effects and improve the eﬃcacy, PegIFN-α-2α has been developed with one dose per week prescription [3]. Previous studies have shown that Peg-IFN-α2a is superior to IFN-α and lamivudine in the treatment of chronic hepatitis B infection [4]. The use of Peg-IFNα-2a has the advantages of limited treatment course and absence of viral resistance, which allows the use of other antiviral agents in early non-responders. Several studies have also shown that there were a combination of virological factors, host immunological factors, and genetic factors attribute to the response of hepatitis B virus to Peg-IFN-α-2a [5][6][7]. Because no antiviral treatment is perfect, early and accurate prediction of treatment response is helpful to modify therapeutic regimen based on these indications [8]. Pretreatment predictors can help physicians to select the best time of initiation as well as the type of medication to be administered. Correct choice of the ﬁrst-line potent therapy is essential to achieve long-term SVR. Therefore, identifying host-dependent and virus-related factors would signiﬁcantly predict sustained response to IFN in patients with chronic hepatitis B disease.

## 2. Objectives

The purpose of this study was to determine whether the type I IFN-α receptor β subunit (IFNAR2) expression in liver and HBV genotypes inﬂuence the response to PegIFN-α-2a (Pegasus, Roche, Basel, Switzerland) immunotherapy in chronic hepatitis B disease.

## 3. Patients and Methods

### 3.1. Subjects

Outpatients from 161 hospital of PLA and Nanfang Hospital of Southern Medical University with chronic hepatitis B disease were screened and included in this study. Our study enrolled 65 eligible patients with chronic hepatitis B infection of which ﬁve patients did not ﬁnish the treatment as a result of severity of side-effects. Exclusion criteria included patients with infections caused by other viruses, such as hepatitis A, C, D, and E viruses, cytomegalovirus, Epstein-Barr virus, and human immunodeﬁciency virus, and patients with autoimmune hepatitis, primary biliary cirrhosis, sclerosing cholangitis, metabolic disorders, liver cirrhosis, decompensated liver diseases, a current or past history of alcohol abuse, psychiatric conditions, previous liver transplantation, or evidence of hepatocellular carcinoma, and patients who were taking antiviral drugs or interferon before the biopsy. Patients fulﬁlling the inclusion and exclusion criteria were offered to participate in the study. Those patients who opted for antiviral therapy started 180 μg Peg-IFN-α-2a subcutaneously per week for 48 weeks and followed up for 24 weeks. At the end of treatment, response to Peg-IFN-α-2a therapy was deﬁned as normalization of serum ALT levels and HBV DNA loss. Continuous response after cessation of treatment in the patients was deﬁned as sustained viral response (SVR). Relapse is deﬁned as undetectable HBV-DNA level in serum and normal ALT at the end of the treatment, but detectable HBV-DNA level in serum or abnormal ALT at the end of follow-up. Non-responders were deﬁned as those with relapse and those who did not attain viral or biologic response at the end of treatment. The study protocol was approved and monitored by the ethics committee of Nanfang and 161 Hospital, and written informed consent was obtained from the patients.

### 3.2. Serological Tests

Sera HBeAg versus anti-HBeAg and HBsAg versus anti-HBsAg were measured using AxSYM MEI kits (Abbott laboratories). The serum HBV DNA level was determined using Roche real-time light cycle ﬂuorescence PCR with a lower limit of detection of 1 × 103 copies/mL. Serum ALT levels were measured using commercial kits by a Hitachi Automatic Biochemical Analyzer. The remaining of sera were stored at -70 ℃.

### 3.3. Histological Evaluation

All liver biopsies were performed percutaneously using a 16-gauge needle without or with radiological/ultrasound guidance. Liver biopsy specimens were retrospectively reviewed by an experienced hepatopathologist blinded to patients and clinical status. The liver inﬂammatory grade and ﬁbrotic stage were evaluated according to modiﬁed Knodell histological activity index [9]. Histological extent of liver injury was classiﬁed according to the involved area of tissue necrosis and inﬂammation: G0 no inﬂammation, G1 mild inﬂammation, G2 hepatocellular damage or moderate inﬂammation, G3 severe hepatocellular damage, G4 severe and widespread hepatitis with bridging conﬂuent necrosis.

### 3.4. Genotyping of HBV

HBV DNA samples extracted from patients sera were subjected to polymerase chain reaction (PCR) ampliﬁcation. PCR reactions were run by using HBV S gene type-speciﬁc primers and then PCR products were subjected to restriction fragment – length polymorphism (RFLP) analysis as previously described [10][11][12].

### 3.5. IFNAR2 Expression in Liver

The expression of IFNAR2 in the liver was detected by the use of Strept – Avidin–Biotin–Peroxidase Complex (SABC) immune histochemical staining methods. All the slides were allowed to dry at room temperature and rinsed twice with phosphate buffered saline (PBS) solution (pH 7.2), then incubated with blocking solution 3 % of H2O2 for 10 min at room temperature. Non-speciﬁc antibody binding was blocked by 1.5 % rabbit serum albumin in PBS solution for 20 min. Slides were incubated overnight at 4℃ with rabbit anti-human IFNAR2 polyclonal antibody( R and D, Minneapolis ,USA), diluted in 1:1000 in PBS. The bridging antibody (biotin-labeled goat anti-rabbit IgG) and SABC complex were diluted to 1:100 and incubated with the specimens for 20 min at room temperature. Finally color development was achieved by the use of diaminobenzidine tetrachloride and the slides were counterstained with hematoxylin. Positive rate (brown color cells) was automatically measured with the biological image analysis system 2000 (Leica, Berlin, Germany).

### 3.6. Statistical Analysis

The clinical quantitative data were reported as mean ± SD, and categorical variables were expressed together with frequency and percentages. The intergroup differences of quantitative variables were veriﬁed by Student’s t-test, while the differences of categorical values were analyzed by χ2 test. A multivariate analysis with a forward stepwise logistic regression model including all factors that had a P value of 0.05 in univariate analysis was then performed to identify factors predict an SVR, independently. Odds ratio (OR) with 95 % conﬁdence interval (95 % CI) was calculated. All analyses were conducted using SPSS version 15.0 (SPSS, Chicago, IL, USA). P-values were set at 0.05 (P < 0.05) for statistically signiﬁcant difference.

## 4. Results

### 4.1. Characteristics of the Patients

**Figure 1:**
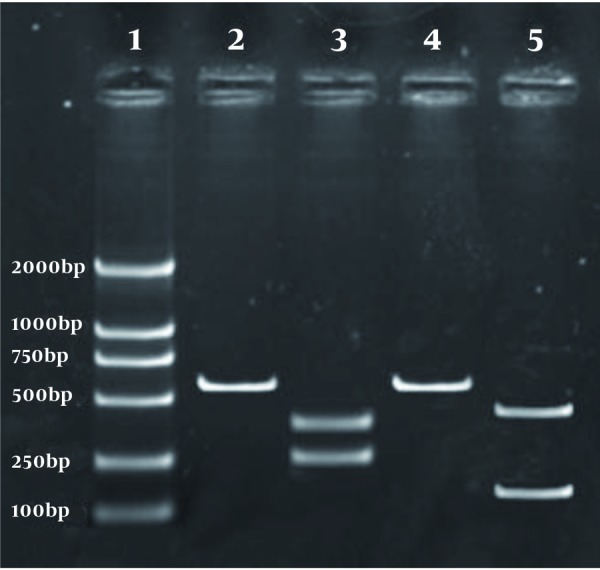
PCR-Based RFLP Analysis of HBV Genotypes (Lane 1: DNA size markers (100, 250, 500, 700, 1000, 2000 bp), Lane 2: undigested B genotype PCR product, Lane 3: StyI digestion patterns in B genotype (253bp and 332bp), Lane 4: undigested C genotype PCR product, Lane 5: BsrI digestion patterns in C genotype (126bp and 459bp))

65 patients were enrolled and received peg-IFN-α-2a. Of these patients, ﬁve did not completed treatment because of severe adverse effects, such as depression (n = 2), irritability (n = 1), thyroiditis (n = 2); thus 60 patients who completed the study were included in the ﬁnal analysis. Distribution of HBV genotypes in patients with chronic hepatitis B disease and base-line characteristics of HBV DNA genotypes were detected with PCR-RFLP and shown in Figure 1. Distribution of different HBV genotypes in patients with chronic hepatitis B disease was shown in Table 1. Genotype B was found in 33 patients (55.0 %), genotype C in 19 (31.7 %), mixed genotype (B + C) in three (5.0 %), genotypes D in three (5.0 %), and two subtypes were not recognized. Genotypes B and C were distinguished as major genotypes in this area. The baseline clinical data of patients with HBV genotypes B and C were compared in Table 2. No signiﬁcant differences in male: female ratio, mean age, baseline ALT, and HBV DNA levels was observed between the two groups when classiﬁed by HBV genotype.

**Table 1:** Distribution of Different Hepatitis B Virus Genotypes in Patients With Chronic Hepatitis B

	**Patients, No. (%)**
B	33 (55.0)
C	19 (31.7)
B + C	3 (5.0)
D	3 (5.0)

**Table 2:** Baseline Characteristics of 52 Patients With Chronic Hepatitis B Virus Infection, Classified by Hepatitis B Virus Genotypes

	**HBV a Genotype B (n = 33)**	**HBV Genotype C (n=19)**	***P value***
Gender, No.			NS a
Male	23	10	
Female	10	9	
Age, y, mean ± SD	31.5 ± 7.5	31.6 ± 9	NS
ALT a level, U/L, mean ± SD	163.5 ± 97.5	134.0 ± 59.6	NS
HBeAg a positivity, No. (%)	20 (60.6)	14 (73.7)	NS
HBV DNA levels, log10copies/ml, mean ± SD	9.3 ± 3.1	9.1 ± 1.9	NS

^a^ Abbreviations: ALT: Alanine Aminotransferase, HBeAg: Hepatitis B e Antigen, NS: Not Significant

### 4.2. Response of HBV Genotypes B and C to Peg-IFN-α-2a Therapy

Patients with HBV genotype C exhibited signiﬁcantly lower SVR to Peg-IFN-α-2a, only 26.3 %, whereas 66.7 % of patients with genotype B manifested SVR to Peg-IFN-α-2a (P = 0.009) (Table 3).

**Table 3:** Response to Interferon-α Therapy in Patients With Different Genotypes

	**SVR **a**, No.**	**NSVR **a**, No.**	**Total, No.**	***P* value**
B	22	11	33	0.009
C	5	14	19	0.009

^a^ Abbreviations: NSVR, not sustained viral response; SVR, sustained viral response

### 4.3. IFNAR2 Protein Expression Correlates With Liver Inﬂammatory Grade

**Figure 2:**
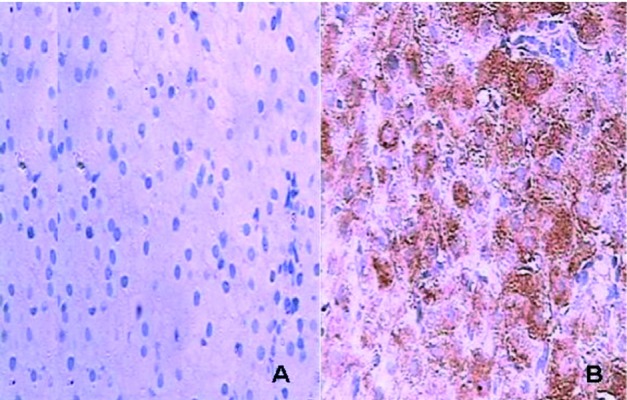
Immunostaining of IFNAR2 in Liverz (A: No background staining was observed in the negative control experiments B: IFNAR2 expressed in cytoplasm and cytomembrane of hepatocytes)

IFNAR2 detected mainly in cytoplasm or cytomembrane of hepatocytes was shown in Figure 2. The IFNAR2 gray scale in G1, G2, and G3 were (5.34 ± 1.66), (6.52 ± 1.56), and (26.68 ± 4.94), respectively. There was a signiﬁcant difference between G1 and G3, or G2 and G3 (P < 0.001).

### 4.4. SVR to Peg-IFN-α-2a Therapy and Factors Affecting SVR

Overall, 30 patients (46.2 % by intention-to-treat analysis and 50.0 % by per protocol analysis) achieved SVR. Only those who completed the treatment were included for further analyses to identify factors affecting SVR. Patients with high IFNAR2 protein expression showed a signiﬁcant impact on patient’s SVR to Peg-IFN-α-2a therapy (P = 0.028). The baseline clinical data of 60 patients with chronic hepatitis B infection are compared in Table 3. No signiﬁcant differences in male: female ratio, mean age, baseline ALT, and HBV DNA levels was observed between the two groups when classiﬁed by HBeAg status. Patients with HBeAg positive sera exhibited signiﬁcantly lower SVR to Peg-IFN-α-2a compared to serum negative HBeAg patients (33.3 % vs. 61.1 %, P = 0.035) (Table 4). Baseline factors associated with SVR suc h as IFNAR2 expression in the liver, ALT level, gender, age, HBeAg status, HBV genotype, and HBV DNA level were included in multiple logistic regression analysis model. This model identiﬁed three variables independently associated with increased SVR. They include IFNAR2 expression in the liver (OR = 3.80; 95 % CI: 2.54-5.70; P < 0.05), HBV-DNA level < 105copies/mL (OR = 1.70; 95 % CI: 1.14-2.56; P < 0.05), and ALT level (OR = 1.05; 95 % CI: 1.03-1.08; P = 0.036) (Table 5).

**Table 4:** Baseline Characteristic and Sustained Viral Response to Peg-IFN-α-2a of 60 Patients With Chronic HBV Infection, Classified by HBeAg Status

	**HBeAg a Negative (n = 36)**	**HBeAg Positive (n = 24)**	**P value**
Gender, No.			NS a
Male	23	13	
Female	13	11	
Age, y, mean ± SD	32.5 ± 7.0	31.8 ± 9.5	NS
ALT a level, U/L, mean ± SD	164.5 ± 90.3	136.0 ± 79.5	NS
HBV DNA levels, log10copies/ml, mean ± SD	9.4 ± 3.0	9.2 ± 2.9	NS
SVR a	22.36	8.24	0.035

^a^ Abbreviations: ALT, alanine aminotransferase; HBeAg, Hepatitis B e Antigen; NS, Not Significant; SVR, Sustained Viral Response

**Table 5:** Independent Predictors of Sustained Viral Response Identified by Multivariate Analysis

	**OR (95 % CI)**	***P *value**
IFNAR2	3.80 (2.54-5.7)	< 0.05
HBV DNA level < 10^5^ copies/ml	1.75 (1.14-2.56)	< 0.05
ALT level	1.05 (1.03-1.08)	< 0.05

## 5. Discussion

Chronic hepatitis B is a potentially severe form of liver disease that often progresses to cirrhosis, hepatic decompensating, and hepatocellular carcinoma [13]. Active-viral replication is always associated with rapid disease progression. As a consequence, optimal therapy with antiviral drugs may achieve maximal virologic suppression to improve the clinical outcome. The present study aimed to investigate the role of HBV genotypes and IFNAR2 in response to Peg-IFN-α-2a in Chinese patients with hepatitis B. Until now, based on an intergroup divergence of 8 % or more in the complete nucleotide sequence, HBV can be classiﬁed into nine genotypes (A-I) [14][15][16][17]. It has been shown that HBV genotypes have a characteristic geographic distribution [18]. In addition, several lines of evidence indicate that there are different sustained responses to conventional interferon in patients with different genotypes. Studies suggested that genotype A is associated with a higher rate of IFN-α-induced HBeAg seroconversion than genotype D [18], and genotype B is associated with a higher rate of IFN-α-induced HBeAg clearance than genotype C [19]. Similarly, our study revealed that HBV genotype C was associated with a lower rate of antiviral response to Peg-IFN-α-2a treatment in Chinese patients with chronic hepatitis B disease compared to genotype B (26.3% vs. 66.7 %, P = 0.009). Further studies with enlarged sample sizes are needed to validate our results and the prevalence of mixed HBV genotype B and C infections and their implications for prognosis and treatment. In addition to HBV genotype factor, our study investigated whether expression levels of IFNAR2 in the liver of chronic hepatitis B patients are correlated with the response to Peg-IFN-α-2a therapy. IFNs are known to bind to receptors on the cell surface that facilitate activation of Janus kinase/signal transduction pathway that result in activation of other transcription pathways [20][21]. IFNAR mRNA expression in liver of patients with chronic hepatitis B infection has been shown to be related to the response to IFN-α therapy. Absent or low intrahepatic IFNAR mRNA levels are related to a poor response to IFN-α [22][23]. Our study demonstrated that expression of IFNR2 protein had a close correlation with hepatic inﬂammation. Moreover, the majority of responders with chronic hepatitis B exhibited a high level of IFNR2 protein expression prior to treatment, which conforms to other previous studies [24][25]. To the best of our knowledge, this is the ﬁrst study performed in China to determine both viral genotype and expression level of host IFNR2 that are closely correlated with the response to Peg-IFN-α-2a therapy in patients with chronic hepatitis B disease. In addition to IFNAR2, there are numerous genes involved in interferon-alpha pathway. Future studies are needed to identify those genes involved in Peg-IFN-α-2a therapy. HBeAg-negative in chronic hepatitis B disease usually represents at later phases of disease progression. HBeAg clearance may follow an exacerbation of hepatitis manifested by ALT level elevation [26]. Progression to cirrhosis or liver cancer was frequently observed in these patients [27]. Antiviral treatment, either by long-term nucleoside– nucleotide analogs or administration of IFN, is therefore necessary to inhibit course of disease. However, viral responses to standard IFN or nucleoside–nucleotide therapy were occurred in low rates among these patients. In our study, SVR to Peg-IFN-α-2a was achieved in approximately 50% of HBeAg-negative patients, with a signiﬁcant rates compared to HBeAg-positive patients (33.3 % vs. 61.1 %). Therefore, Peg-IFN is a better choice for HBeAg-negative patients.

In conclusion, HBV genotype B and high expression of IFNAR2 in liver are closely associated with better response to Peg-IFN-α-2a therapy among Chinese patients with chronic hepatitis B disease. Therefore, recognition of these patients before suitable antiviral therapy is very important to judge on the treatment outcome and prognosis.
